# Can whole genome sequencing resolve taxonomic ambiguities in fungi? The case study of *Colletotrichum* associated with ferns

**DOI:** 10.3389/ffunb.2025.1540469

**Published:** 2025-02-28

**Authors:** Andrea Menicucci, Salvatore Iacono, Madalena Ramos, Chiara Fiorenzani, Natalia Aparecida Peres, Lavern Wayne Timmer, Antonio Prodi, Riccardo Baroncelli

**Affiliations:** ^1^ Department of Agricultural and Food Sciences (DISTAL), University of Bologna, Bologna, Italy; ^2^ LEAF - Linking Landscape, Environment, Agriculture and Food Research Centre, School of Agriculture, University of Lisbon, Lisbon, Portugal; ^3^ Plant Pathology Department, Gulf Coast Research and Education Center, University of Florida, Wimauma, FL, United States; ^4^ Plant Pathology Department, Citrus Research and Education Center, Lake Alfred, FL, United States; ^5^ Center for Studies on Bioinspired Agro-Enviromental Technology, University of Naples Federico II, Portici, Italy

**Keywords:** MLST, phylogenomics, WGS, orthologous genes, fungal genomes

## Abstract

**Introduction:**

The genus *Colletotrichum* comprises numerous fungal species with diverse ecological roles, including plant pathogenic, endophytic, and saprophytic lifestyles. Accurate species identification is crucial for understanding host-pathogen interactions, disease epidemiology, and fungal ecology. However, taxonomic classification within *Colletotrichum* remains challenging due to genetic complexity and phenotypic plasticity. Conventional approaches such as single-gene analyses and multilocus sequence typing (MLST) frequently fail to resolve closely related taxa, leading to misidentifications that hinder species delimitation and comparative evolutionary studies. Whole-genome sequencing (WGS) offers a promising alternative by providing genome-wide resolution for phylogenetic analysis. This study investigates *Colletotrichum* isolates associated with the fern *Rumohra adiantiformis* and evaluates the effectiveness of WGS in addressing taxonomic uncertainties.

**Methods:**

A total of 18 *Colletotrichum* isolates, including fern-associated strains, were analyzed. Genomic DNA was extracted and sequenced using the Illumina NovaSeq platform. High-quality genome assemblies were generated, and gene prediction was conducted using AUGUSTUS. Orthogroup assignment and phylogenomic analysis were performed based on single-copy orthologs, and phylogenetic trees were constructed using MLST and WGS-based approaches. Comparative analyses were carried out to assess the taxonomic resolution provided by WGS in relation to traditional methods.

**Results:**

Genome-wide phylogenomic analysis revealed distinct evolutionary lineages among *Colletotrichum* isolates that MLST failed to resolve, highlighting host and geographic differentiation. High-quality genome assemblies were obtained, with 98.3% of genes assigned to orthogroups, indicating strong genomic conservation. Phylogenetic analyses confirmed a close relationship between *Rumohra adiantiformis*-associated isolates and *Colletotrichum filicis*, reinforcing the effectiveness of WGS in species identification.

**Discussion:**

These findings demonstrate the superiority of WGS over MLST in resolving species boundaries and reconstructing evolutionary relationships. The enhanced resolution provided by genome-wide data enables more accurate taxonomic classification, reducing misidentifications and improving our understanding of fungal biodiversity. By refining *Colletotrichum* taxonomy, WGS facilitates ecological and pathogenic studies, offering a robust framework for future research in fungal systematics and plant pathology. As sequencing technologies continue to advance, WGS is expected to become a standard tool for fungal species delineation and evolutionary studies.

## Introduction

*Colletotrichum* is a genus of ascomycete fungi widely recognized for its role as a significant plant pathogen, responsible for economically impactful diseases such as anthracnose across numerous crops (Dean et al., 2012). Although *Colletotrichum* species are well-known for causing anthracnose in nearly all cultivated crops worldwide, knowledge remains limited regarding species that infect plants in natural ecosystems (Talhinhas and Baroncelli, 2023). Beyond its economic relevance, *Colletotrichum* also serves as an important model for investigating plant-pathogen interactions, offering insights into mechanisms of host adaptation and virulence (Perfect et al., 1999; Baroncelli et al., 2017). One factor contributing to the interest in *Colletotrichum* is the genus’ diverse range of lifestyles. Some species function as hemibiotrophic pathogens (O’Connell et al., 2012), initially surviving on living tissue and later transitioning to kill it, while others act as endophytes or saprophytes (Jayawardena et al., 2021). This versatility in infection strategies, together with considerable genetic variability, presents substantial challenges for crop protection and disease management (da Silva et al., 2020).

Taxonomy plays a fundamental role in the study of plant pathogens, serving as the foundation for understanding the diversity, biology, and evolutionary relationships of these organisms. Accurate taxonomic classification is essential for linking specific biological behaviors, such as pathogenicity, host specificity, and infection strategies, to particular species or lineages, as well as for reconstructing evolutionary patterns and relationships.

In plant pathology, identifying pathogens to the species or even subspecies level allows researchers to predict potential host ranges, assess disease risks, and design targeted management strategies. For example, in genera like *Colletotrichum*, where species exhibit diverse lifestyles (e.g., pathogenic, endophytic, or saprophytic) (Jayawardena et al., 2021), precise taxonomy becomes essential to distinguish between harmful and benign strains. Misclassification can lead to incorrect assumptions about a species’ pathogenicity or ecological role, hindering effective disease management and potentially leading to unnecessary treatments or overlooked threats. Advances in taxonomy, often driven by molecular tools, enable researchers to refine species boundaries, thus linking specific biological behaviors more accurately to taxonomic groups. This link is critical not only for understanding the evolutionary dynamics of plant-pathogen interactions but also for developing sustainable and effective control measures tailored to each pathogen’s unique characteristics. The recent clarification of *Colletotrichum* taxonomy, which now includes over 340 recognized species grouped into 20 complexes, has facilitated a better understanding of the diversity and host association patterns of these fungi (Talhinhas and Baroncelli, 2023)​. Some species, such as *C. fructicola*, infect over 50 plant species, including economically important crops like strawberries, apples, and pears, leading to significant economic losses (Liang et al., 2018). In contrast, other species, like *C. lupini*, exhibit strong host specificity (Baroncelli et al., 2016). Having said that, species delimitation in the fungal genus *Colletotrichum* is notoriously difficult due to the paucity of distinctive morphological characters and the plasticity of their characters under different conditions. Traditionally, *Colletotrichum* species were identified based on morphology and host specificity, which led to the description of hundreds of species, many of which were later found to be synonyms (Talhinhas and Baroncelli, 2021). Attempts to clarify the taxonomy of the genus using single-gene phylogenetic analyses have been unsuccessful. Sequence data from a single gene may not provide sufficient resolution to distinguish closely related species. This is because individual genes can have different evolutionary histories not reflecting the species relationships (Lücking et al., 2020). The usage of single genetic markers is also inefficient, partly due to the high level of misidentification in public sequence databases such as GenBank (Chorlton, 2024). This taxonomic confusion has hindered efforts to understand host-pathogen relationships, accurately diagnose diseases, develop effective control strategies, and establish cost-effective quarantine programs. Multilocus sequence typing (MLST) is a widely used technique in microbiology for studying evolutionary relationships and delimiting species. It is based on the idea that housekeeping genes, essential for cellular survival, evolve at a relatively constant rate (Urwin and Maiden, 2003). MLST has been successfully applied to species delimitation in a variety of fungal groups, including *Colletotrichum* (Marín-Felix et al., 2017). While MLST has provided a useful approach to species delimitation in *Colletotrichum* (Jayawardena et al., 2016, 2020, 2021), it is essential to recognize its limitations. Different *Colletotrichum* complexes often require distinct sets of gene markers, making comparisons across complexes inconsistent and less reliable. An illustrative example is the work of MacKenzie and colleagues (MacKenzie et al., 2009), who used phylogenetic analysis to explore the relationships between isolates from the acutatum species complex, collected from various fruit crops and fern in Florida. For this purpose, the internal transcribed spacer (ITS) region, a partial sequence of the glyceraldehyde-3-phosphate dehydrogenase (GAPDH) gene, and the glutamine synthetase (GS) gene were employed. These sequences were used to construct phylogenetic trees, revealing genetic similarity among isolates from the same host, while showing clear differences from isolates derived from other hosts. An interesting exception was observed with isolates from ferns, which showed distinct and non-concordant clustering patterns in the GAPDH and GS gene trees. Genealogical concordance, or the agreement in tree topology among gene trees, is expected when genes share a common evolutionary history. However, in this case, the GAPDH and GS trees revealed different topologies for fern isolates, suggesting that these genes may reflect different evolutionary histories or processes.

The rise of next-generation sequencing (NGS) technologies, coupled with decreasing sequencing costs, has opened a transformative era for fungal taxonomy. Central to this shift is the adoption of whole-genome sequencing (WGS), which provides a more comprehensive view of genetic diversity compared to traditional molecular methods. By accessing entire genomes, WGS enables the construction of robust phylogenies based on thousands of genes, offering higher resolution and accuracy in species classification than single- or multiple-genes approaches (Kapli et al., 2020).

One key advantage of WGS is the identification of orthologous genes, which are particularly valuable in phylogenomic studies. Since these genes are inherited from a common ancestor, they provide reliable markers for species divergence, avoiding complications from within-species gene duplication events (Sonnhammer and Koonin, 2002). Advanced tools like Orthofinder and MAFFT are frequently used to identify and align these orthologs, enabling the construction of phylogenetic trees with thousands of genes, offering far greater resolution than single-gene trees (Kapli et al., 2020; Salotti et al., 2023). The broader impact of WGS is evident in the restructuring of the fungal tree of life. Molecular phylogenetic studies have shown that many traditional taxonomic groups are polyphyletic, meaning they contain species without a shared evolutionary ancestor. This has led to significant revisions across taxonomic levels, from phylum down to species (Hyde et al., 2024). One prominent example is the disbanding of the phylum Zygomycota, which molecular evidence revealed to be polyphyletic, resulting in its reclassification into new phyla like Mucoromycota and Zoopagomycota (Borman and Johnson, 2023). Genetic approaches have also resolved long-standing taxonomic issues, such as the distinction between the sexual and asexual stages of fungi. Historically, dual nomenclature was used, assigning separate names to the sexual (teleomorph) and asexual (anamorph) stages of the same species. Molecular evidence has since shown that these stages are genetically identical, leading to the unification of teleomorph and anamorph names under a single species designation, as formalized by the Amsterdam Declaration of Fungal Nomenclature in 2011 (Hawksworth et al., 2011). This shift has simplified fungal taxonomy and reduced confusion around species identification (Borman and Johnson, 2023). Despite the remarkable benefits of whole-genome sequencing (WGS), several challenges remain. One of the primary limitations of this approach is the insufficient availability of genomes to represent all known species and their intraspecific variability. Additionally, the sheer volume of genomic data requires sophisticated computational tools for analysis, which must address complex issues such as incomplete lineage sorting, horizontal gene transfer, and gene duplication. These factors can obscure species relationships and complicate the construction of accurate phylogenies if not properly managed (Kapli et al., 2020). Furthermore, integrating WGS data with ecological and morphological information is still an ongoing challenge, as taxonomic decisions should ideally reflect both genetic and phenotypic diversity.

This study investigates the potential of whole-genome sequencing (WGS) to resolve longstanding taxonomic ambiguities in *Colletotrichum*, a genus known for its diverse ecological roles and pathogenic capabilities. We hypothesize that WGS, by offering access to complete genomic data, will provide significantly higher resolution in fungal isolate characterization compared to traditional multi-locus sequence typing (MLST). Specifically, we propose that WGS will reveal fine-scale genetic differences that remain undetected by MLST, enabling precise differentiation between closely related strains.

Furthermore, we hypothesize that the comprehensive genetic information provided by WGS will not only improve the accuracy of taxonomic identification but also open new avenues for functional genomic studies. By examining the full genetic blueprint of these isolates, we aim to uncover the genetic mechanisms underlying host specificity, pathogenicity, and environmental adaptation in *Colletotrichum* (Baroncelli et al., 2016; Crouch et al., 2014). This approach could facilitate the identification of novel pathogenicity factors and adaptive traits, offering insights into how these fungi interact with their hosts and persist in varying ecological niches. Ultimately, we anticipate that the integration of WGS into fungal taxonomy and functional studies will enhance our understanding of the evolutionary processes shaping the diversity and ecological success of *Colletotrichum* species.

## Materials and methods

### Fungal material

A total of 18 *Colletotrichum* isolates were included in this study. Seven of these isolates (H-8, 05-161, 05-200, H-3, 05-155, H-24, H-19) were retrieved from fern fronds previously collected between USA and Costa Rica (MacKenzie et al., 2009). *Colletotrichum filicis* CBS 101611 (Damm et al., 2012; Crous et al., 2021) is the ex-type the only genome available, and for these reasons was selected as reference genome in our dataset too. Isolates and data collected from each isolate are listed in **Table 1**.

**Table 1:** *Colletotrichum* genomes used in the present study.

Species	Strain	Host	Origin	Reference
*Colletotrichum abscissum*	IMI 504890	*Citrus x sinensis*	USA	Baroncelli et al., 2024
*Colletotrichum costaricense*	IMI 309622*	*Coffea* sp.	Costa Rica	Baroncelli et al., 2024
*Colletotrichum cuscutae*	IMI 304802*	*Cuscuta* sp.	Dominica	Baroncelli et al., 2024
*Colletotrichum filicis*	CBS 101611*	unclassified Pteridophyta	Costa Rica	Goulin et al., 2023
*Colletotrichum limetticola*	KLA-Anderson	*Citrus × aurantiifolia*	USA	Menicucci et al., 2023
*Colletotrichum lupini*	CBS 109225*	*Lupinus albus*	Ukraine	Baroncelli et al., 2024
*Colletotrichum lupini*	IMI 504893	*Lupinus albus*	France	Baroncelli et al., 2021
*Colletotrichum melonis*	CBS 134730	*Malus domestica*	Brazil	Baroncelli et al., 2024
Colletotrichum nymphaeae**	IMI 504889	*Fragaria x ananassa*	Denmark	Baroncelli et al., 2016
*Colletotrichum paranaense*	IMI 384185	*Caryocar brasiliense*	Brazil	Baroncelli et al., 2024
*Colletotrichum tamarilloi*	CBS 129955	*Solanum betaceum*	Colombia	Baroncelli et al., 2024
***Colletotrichum* sp.**	H-24***	***Rumohra adiantiformis***	**Costa Rica**	**This work**
***Colletotrichum* sp.**	H-19***	***Rumohra adiantiformis***	**Costa Rica**	**This work**
***Colletotrichum* sp.**	H-8***	***Rumohra adiantiformis***	**Costa Rica**	**This work**
***Colletotrichum* sp.**	05-161***	***Rumohra adiantiformis***	**USA**	**This work**
***Colletotrichum* sp.**	05-200***	***Rumohra adiantiformis***	**USA**	**This work**
***Colletotrichum* sp.**	H-3***	***Rumohra adiantiformis***	**Costa Rica**	**This work**
***Colletotrichum* sp.**	05-155***	***Rumohra adiantiformis***	**USA**	**This work**

In bold are reported the isolates sequenced and characterized in this study.

*Ex-type or holo-type. **Colletotrichum nymphaeae was selected as outgroup. ***Isolates originally described by MacKenzie et al., 2009.

### DNA extraction

All isolates sequenced in this work were grown on PDA plates for seven days before DNA extraction. The fungal mycelium was scraped from the surface of a PDA plate using a sterile scalpel and transferred to a sterile 1.5 mL tube. Genomic DNA was extracted from the isolates using a modified CTAB method (Prodi et al., 2011).

Mycelium was ground by adding 700 μL of 3% CTAB solution using the Motor-Driven Tissue grinder G50 (Coyote Bioscience Company, Beijing, China). The tubes were placed in a water bath at 65°C for 30 minutes and then centrifugated at 13,225 x g for 10 minutes. 500 μL of the supernatant was transferred in a new sterile 1.5 mL tube and 500 μL of isoamyl alcohol-chloroform (1:24) was added and mixed. After centrifugation at 13,225 x g for 10 minutes, the supernatant was transferred into a new sterile tube. DNA was precipitated by adding an equal volume of ice-cold iso-propanol. The samples were incubated overnight at -20°C. The DNA was centrifuged at 13,225 x g at 4°C for 10 minutes and the isopropanol was removed. Two washes of the pellet were then performed by the addition of 500 μL of 70% ethanol and a subsequent centrifugation at 13,225 x g at 4°C for 5 minutes. The DNA was resuspended in 50 μL of sterile nuclease-free water, quantified, and assessed for quality using a NanoDrop ND-1000 spectrophotometer (Thermo Scientific, DE, USA). DNA was then stored at -20°C before sequencing.

### Genome sequencing, assembly and gene prediction

**Figure 1:**
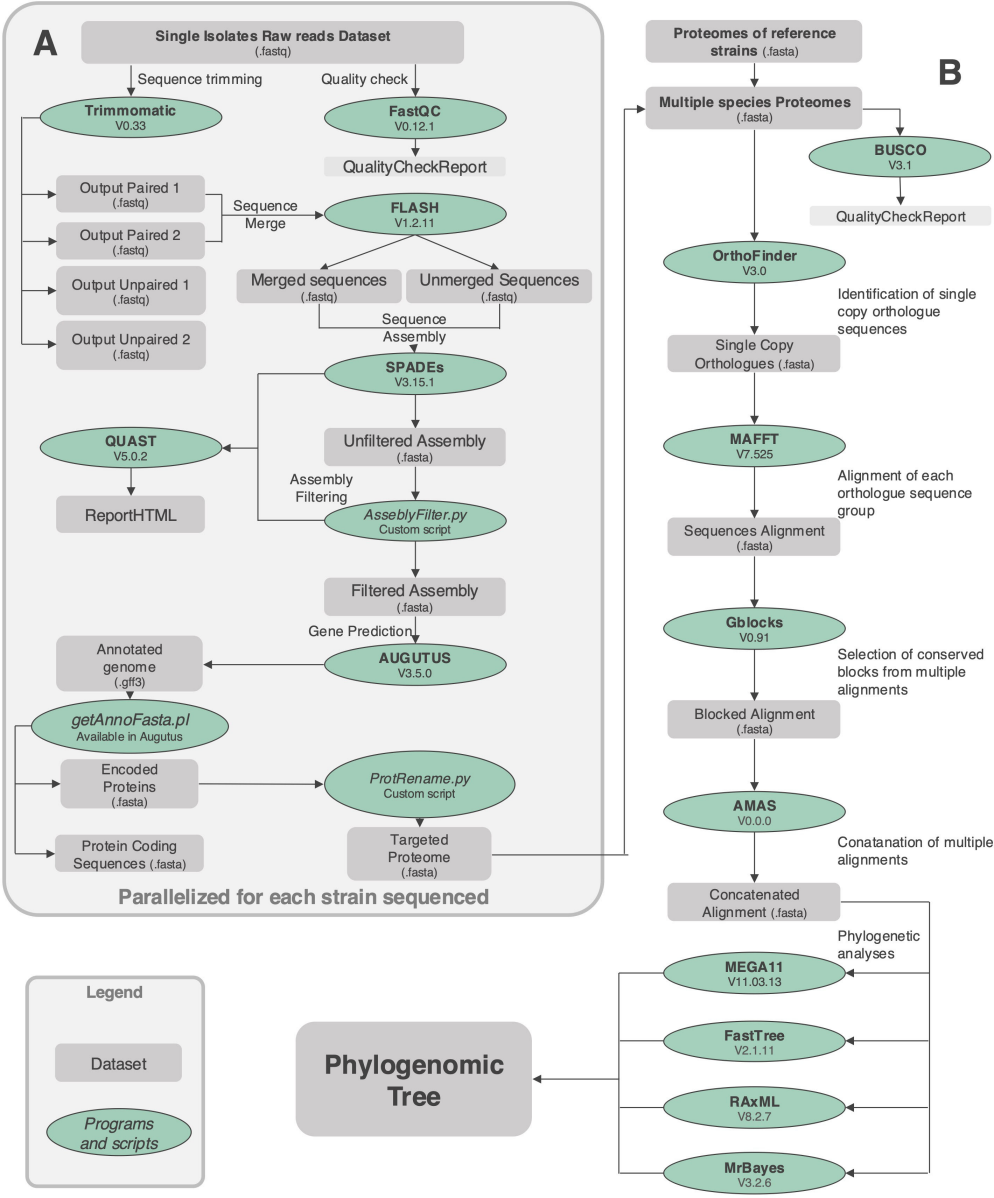
Workflow illustrating the bioinformatic approach used to characterize the strains isolated from fern in this study. The pipeline encompasses key steps such as quality control, read preprocessing, genome assembly, and annotation to extract coding sequences and proteomes **(A)**. Orthogroups identification, alignment of single-copy orthologues, and phylogenomic tree construction were conducted to infer evolutionary relationships **(B)**. Custom scripts and rigorous quality checks were integrated throughout the workflow to ensure reliable and consistent results. This approach enabled the comprehensive characterization of fungal strains and their evolutionary analysis.

The library preparation and genome sequencing of the Colletotrichum genomes were both performed by an external service provider, Personal Genomics, located at Via Roveggia 43b, 37136, Verona, Italy. The sequencing was carried out using the Illumina NovaSeq 6000 platform with a 150 bp paired-end configuration. The quality of the reads was evaluated using FastQC v0.12.1 (Babraham Bioinformatics, Cambridge, UK). Sequence adapters and low-quality reads were trimmed with Trimmomatic v0.33 (Bolger et al., 2014). Pair-end reads were merged with FLASH v1.2.11 (Magoč and Salzberg, 2011). Merged and unmerged reads were then assembled using SPAdes v3.15.1 (Bankevich et al., 2012). Scaffolds with low coverage were removed as possible contaminations. The completeness of the assembly was assessed using BUSCO v3.1 (Simão et al., 2015) while statistics were evaluated with QUAST v5.0.2 (Gurevich et al., 2013). Gene prediction was performed using AUGUSTUS v3.5.0 (Stanke et al., 2006). We use data produced by previous work (Baroncelli et al., 2024) to build the gene model for the closely related species *Colletotrichum lupini*; RNAseq data publicly available (SRX2782478) were assembled using rnaSPAdes v3.15.1 (Bushmanova et al., 2019), assembled transcripts were used to train the genome assembly (GCA_030913515.1) through AUGUSTUS v3.5.0 (Stanke et al., 2008). To extract protein-coding sequences from the GFF3 output, we used a pre-configured Perl script, getAnnoFasta.pl, provided with the AUGUSTUS v3.5.0 package. Finally, a custom script, (ProtRename.py available at https://github.com/RiccardoBaroncelli) was employed to rename proteomes file, streamlining downstream analysis. The details of the bioinformatic approach employed are illustrated in the workflow shown in **Figure 1A**.

### MLST alignment and phylogenetic analysis

For each genome, the final assembly was imported in Geneious Prime v2024.0.2 (https://www.geneious.com) where a local database was created. BLAST analyses were performed on the local databases using as query reference sequences for each *locus*. Seven *loci* were retrieved and used for phylogenetic analysis: the internal transcribed spacer (ITS) region, a partial sequence of the glyceraldehyde-3-phosphate dehydrogenase (GAPDH) gene, the glutamine synthetase (GS) gene, the partial sequence of the beta-tubulin 2 (TUB2) gene, the histone-3 (HIS-3), the chitin synthetase gene (CHS-1) and actin (ACT) (Damm et al., 2012). The sequences of the seven genes of each analyzed genomes were extracted using the ‘extract reads’ plug-in. These sequences were then aligned using MAFFT v7.525 (Katoh and Standley, 2013) and manually adjusted, where necessary, with Geneious Prime v.2024.0.2. The multiple sequence alignments were exported to MEGA11 (Tamura et al., 2021), where the optimal substitution model for each individual dataset was calculated. The multi-locus alignment was performed using Geneious Prime v2024.0.2. Phylogenetic analyses were conducted using Randomized Axelerated Maximum Likelihood (ML) (Stamatakis, 2006), Bayesian Inference (BI) (Ronquist and Huelsenbeck, 2003), and Approximate Maximum Likelihood (Price et al., 2010) for each gene and for the concatenated genes. Maximum likelihood analyses were constructed with the RAxML v8.2.7 software (Stamatakis, 2014) using the GTR CAT model with 1,000 bootstrap replicates. A Bayesian phylogenetic analysis was conducted using a Markov Chain Monte Carlo (MCMC) algorithm in MrBayes v3.6.2 (Ronquist et al., 2012), employing the “nst=2” and a rate variation “=equal” parameters. Four Markov Chain Monte Carlo (MCMC) chains were run from random starting trees for 1,000,000 generations, with sampling occurring every 1,000 generations. The initial 25% of the trees generated were discarded as burn-in, and posterior probabilities (PP) were calculated based on the remaining trees (AvgStdDev = 0.006403). For the approximate maximum likelihood analysis, FastTree v2.1.11 (Price et al., 2010) was used with standard settings as implemented in Geneious Prime v2024.0.2. Phylogenetic trees were compared as described by Shimodaira and Hasegawa (1999), Steenwyk et al. (2023) and Tsang et al. (2017).

### Proteome clustering and phylogenomic analysis

To prepare the proteomes, an AUGUSTUS built-in Perl script (getAnnoFasta.pl) was used to extract the CDS and amino acid sequences from the AUGUSTUS output, and a renaming script was applied to standardize the names of the amino acid files for clarity. The phylogenomic analysis performed was based on clustering proteins into orthologous groups using Orthofinder v3.0 (Emms and Kelly, 2019), for the identification of single-copy orthologues. Single-copy orthologues sequences were aligned using MAFFT v7.525 (Katoh and Standley, 2013). To enhance alignment quality, we used Gblocks v0.91 (Castresana, 2000) to eliminate poorly aligned regions and gaps that could introduce noise into the phylogenetic analysis. Gblocks v0.91 automates the trimming process, retaining only conserved regions that are highly informative for phylogenetic reconstruction. After generating and trimming alignments for each aminoacid sequence alignment, we concatenated the individual alignments into a single dataset. To evaluate the quality of these alignments, we employed AMAS (Borowiec, 2016), which computes key alignment statistics, including alignment length and the number of conserved sites. These metrics were used to assess the robustness and informativeness of each alignment. The phylogenomic tree was constructed using MrBayes, FastTree, RAxML and MEGA, as described in the “MLST Alignment and Phylogenetic analysis” section, which inferred the tree through either Bayesian inference or approximate maximum likelihood methods.

The details of the bioinformatic approach employed are illustrated in the workflow shown in **Figure 1B**. This comprehensive pipeline integrated state-of-the-art tools with systematic quality control measures to ensure the generation of high-quality phylogenomic data. Key steps included robust gene prediction, precise orthogroup clustering, and accurate phylogenetic tree inference, all aimed at elucidating evolutionary relationships with high confidence.

## Results

### Genome sequences revealed common features of *Colletotrichum* sp. associated with fern

**Figure 2:**
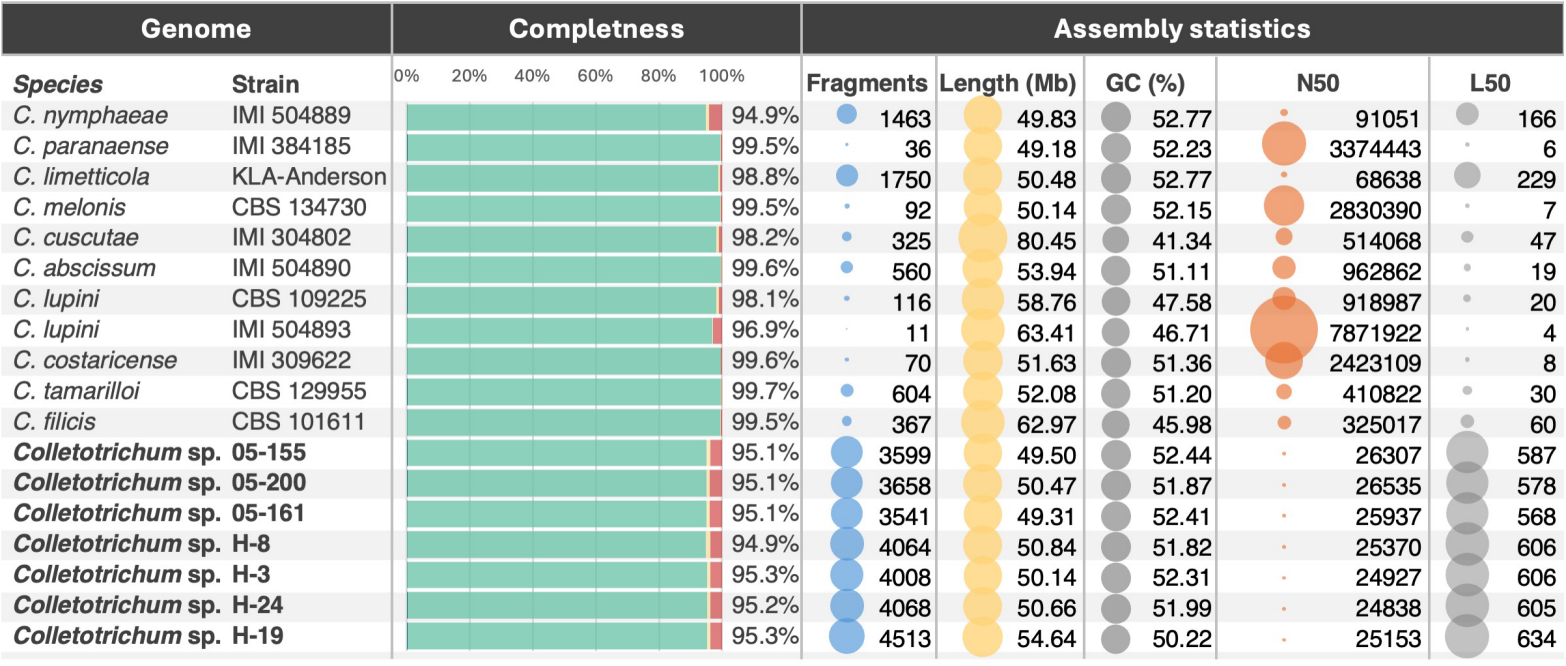
Summary of key metrics for the assembly and annotation of Colletotrichum genomes. The figure illustrates genome completeness using BUSCO scores (middle bar diagram), with percentages representing the following categories: duplicated genes (=D) in blue, single-copy complete genes in green (calculated as the total number of complete single-copy orthologues minus the duplicated ones already assigned =C-D), fragmented genes (=F) in yellow, and missing genes (=M) in red. On the right, additional assembly statistics are visualized as bubble plots, displaying key metrics such as the number of assembly fragments, total assembly length, GC content, N50 values, and L50 values. Genomes sequenced and described in this study are highlighted in bold.

Genome completeness scores, evaluated using BUSCO, indicate a high level of assembly quality across all analyzed genomes and species, with values ranging from 91.5% to 98.4% (calculated as the percentage of Sordariomycetes Single-Copy Orthologs identified in the genomes [C:] **Figure 2**). This high completeness reflects the retention of most single-copy orthologs. All genomes display minimal levels of missing or fragmented BUSCOs ([F:] from 0.3% to 7% and [M:] from 0.1% to 1.5% respectively), emphasizing the robustness of the assemblies. Duplication rates [D:] are generally low, staying below 1% for most isolates. However, a few isolates, notably 05-200, H-3, and H-24, exhibit slightly higher rates of duplication and fragmentation. These deviations may point to subtle strain-specific differences in genome structure, such as gene family expansions or assembly challenges, possibly influenced by repetitive sequences. The assembly statistics were computed using QUAST to assess the quality of the assemblies. The assembly sizes range from approximately 47 Mb to 58 Mb (**Figure 2**), which aligns with the expected genome size for *Colletotrichum* species. Additionally, the GC content remains stable at approximately 51–52%, consistent with previous studies. However, it is important to note that the actual genome size may be larger, and the GC content may be lower, as the genomes were sequenced exclusively using short reads. Consequently, repetitive elements may have collapsed during the assembly process (Talhinhas et al., 2017; Kooij and Pellicer, 2020; Baroncelli et al., 2024). These metrics not only underscore the typical genomic features of the genus but also suggest that the sequenced isolates reflect a diverse representation of *Colletotrichum*’s genetic landscape.

### Single gene phylogenies confirm ambiguities in genealogical concordance

**Figure 3:**
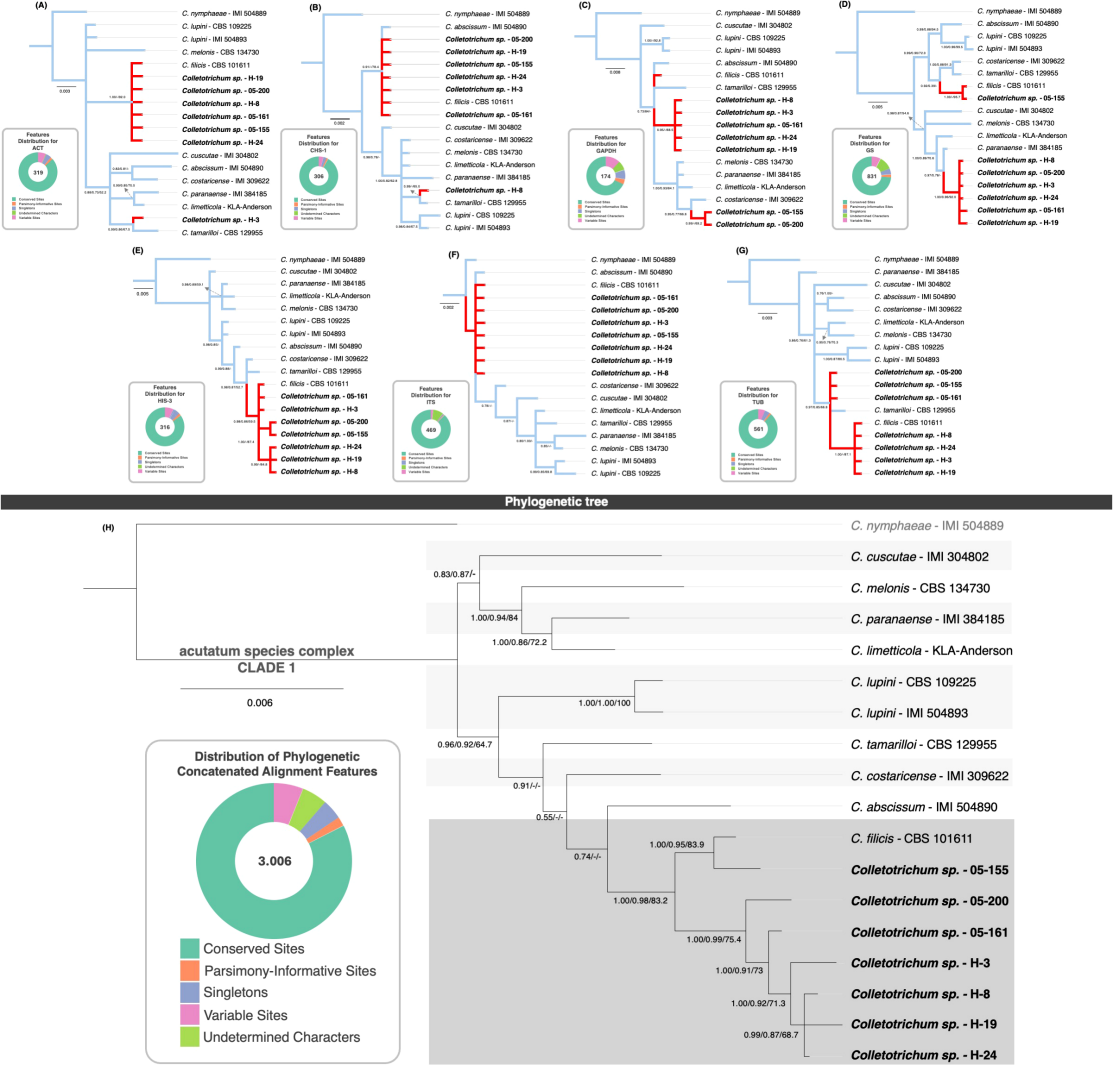
MLST single-gene and concatenated phylogenetic trees of selected isolates. The upper section of the figure presents the phylogenetic trees constructed from individual loci: ACT **(A)**, CHS-1 **(B)**, GAPDH **(C)**, GS **(D)**, HIS-3 **(E)**, ITS **(F)**, and TUB **(G)**. Branches corresponding to *Colletotrichum* sp. isolates sequenced in this study and *C. filicis* CBS 101611 are highlighted in red. To the left of each tree, doughnut charts summarize alignment statistics, illustrating the proportions of conserved sites (dark green), variable sites (pink), parsimony-informative sites (orange), singleton sites (blue), and undetermined characters (light green). The lower section **(H)** displays the concatenated multilocus tree, integrating all seven loci. The cluster containing all *Colletotrichum* sp. isolates and *C. filicis* CBS 101611 is shaded in dark grey. Bootstrap support values derived from MrBayes, FastTree, and RAxML analyses are reported at key nodes. Isolates sequenced in this study are highlighted in bold.

The phylogenetic analyses of single-gene trees (**Figures 3A–G**) reveal diverse topologies and distinct clustering patterns among *Colletotrichum* species, emphasizing the complexity of their evolutionary relationships.

The ACT gene tree (**Figure 3A**) shows that isolate H-3 clusters with *C. tamarilloi*, whereas all other isolates group with *C. filicis*. In contrast, the CHS-1 gene tree (**Figure 3B**) associates isolate H-8 with *C. tamarilloi*, reflecting variability in gene-specific relationships. Meanwhile, the GAPDH gene tree (**Figure 3C**) presents a unique scenario, with isolates 05-155 and 05-200 forming a distinct cluster separate from the remaining isolates, suggesting potential divergence or lineage-specific variation. The GS gene tree (**Figure 3D**) highlights further variability. Here, only isolate 05-155 clusters with *C. filicis*, whereas the remaining isolates form a distinct group closely related to *C. paranaense*. The HIS-3 tree (**Figure 3E**) displays more consistency, with all isolates clustering near and grouping with *C. filicis*. A similar pattern is observed in the ITS gene tree (**Figure 3F**), where the isolates align closely with *C. filicis*, but with *C. abscissum* also appearing within the group, suggesting shared ancestral or conserved sequences. The TUB gene tree (**Figure 3G**) identifies two distinct groups: one composed of isolates from Florida, USA (05-155, 05-161, 05-200) clustering with *C. tamarilloi*, and another including isolates from Costa Rica (H-3, H-8, H-19, H-24) associated with *C. filicis*. These geographic and genetic splits may reflect potential population-level differentiation within the species complex.

None of the individual gene trees fully resolves the observed taxonomic ambiguities. However, trees derived from *loci* such as HIS-3, ITS, and TUB exhibit a closer alignment with the combined tree. An analysis of the correlation between tree resolution and the percentage of variable sites within alignments reveals a nuanced pattern. The ITS region, with the lowest percentage of variable sites (1.9%), demonstrates higher taxonomic concordance, while HIS-3 and TUB, with moderate variability (7.6% and 6.1%, respectively), also produce relatively well-resolved trees. Intermediate variability is observed in ACT and CHS-1 (6.9% and 4.2%, respectively), where *Colletotrichum* sp. mostly cluster with *C. filicis*, apart from one divergent isolate. Conversely, GAPDH and GS alignments, which have the highest variability (14.9% and 9.3%), generate the most divergent tree topologies. These findings suggest that, in this case, *loci* with lower variability are more effective in resolving taxonomic ambiguities compared to those with higher variability. This evidence may suggest that the alignment tools employed in the analysis may face challenges in accurately handling *loci* with high variability, potentially affecting the resolution of taxonomic relationships.

In the concatenated tree based on seven genes (**Figure 3H**), all 17 isolates form a single clade (Clade 1). Within this clade, isolate 05-155 clusters with *C. filicis* CBS 101611, while the remaining isolates are grouped together in a sister clade. The node connecting the sister clade to the group comprising *C. filicis* isolate CBS101611 and isolate 05-155 consistently exhibits high support across all three phylogenetic analyses. Support values are notably robust, with scores of 1.00, 0.98, and 83.2 from MrBayes, FastTree, and RAxML, respectively. However, several nodes show low bootstrap support, and the topologies inferred by the different phylogenetic methods are not always consistent, indicating limited statistical confidence in certain phylogenetic relationships. For example, the topologies generated by FastTree and RAxML reveal slight discrepancies compared to the MrBayes tree. Specifically, the nodes connecting *C. tamarilloi*, *C. costaricense*, and *C. abscissum* to the *C. filicis* cluster lack bootstrap support in the FastTree and RAxML trees. In contrast, the MrBayes tree provides low support values for these nodes (0.55 and 0.74), except for one node, which shows moderately high support (0.91). These limitations make the interpretation of the concatenated tree robust for definitive *Colletotrichum* species identification but challenging for resolving intraspecific evolutionary relationships, highlighting the constraints of multilocus sequence typing (MLST) in addressing certain phylogenetic ambiguities.

These findings underscore the importance of integrating single-gene and concatenated analyses to gain a more comprehensive understanding of *Colletotrichum* species’ evolutionary relationships, while accommodating potential discrepancies among *loci*.

### Phylogenomic analyses clarify the taxonomic designation of newly sequenced isolates and reveal key evolutionary patterns

The phylogenomic analysis was performed using a consensus alignment derived from 7,830 single-copy orthologous sequences across 18 strains. After trimming, the alignment spanned 3,900,074 sites, providing a comprehensive dataset for the study. Notably, only 46 undetermined characters were present, reflecting a near-complete alignment with minimal missing data. The dataset exhibited a high degree of conservation, with 3,711,450 sites conserved across all taxa. Additionally, 188,624 sites were identified as variable, including 28,310 singleton sites and 60,314 parsimony-informative sites, which provided critical phylogenetic signals for reconstructing evolutionary relationships. These metrics underscore the robustness and informativeness of the alignment, ensuring that the phylogenomic tree is based on a statistically solid foundation that captures both conserved regions and evolutionary divergence among the species studied.

**Figure 4:**
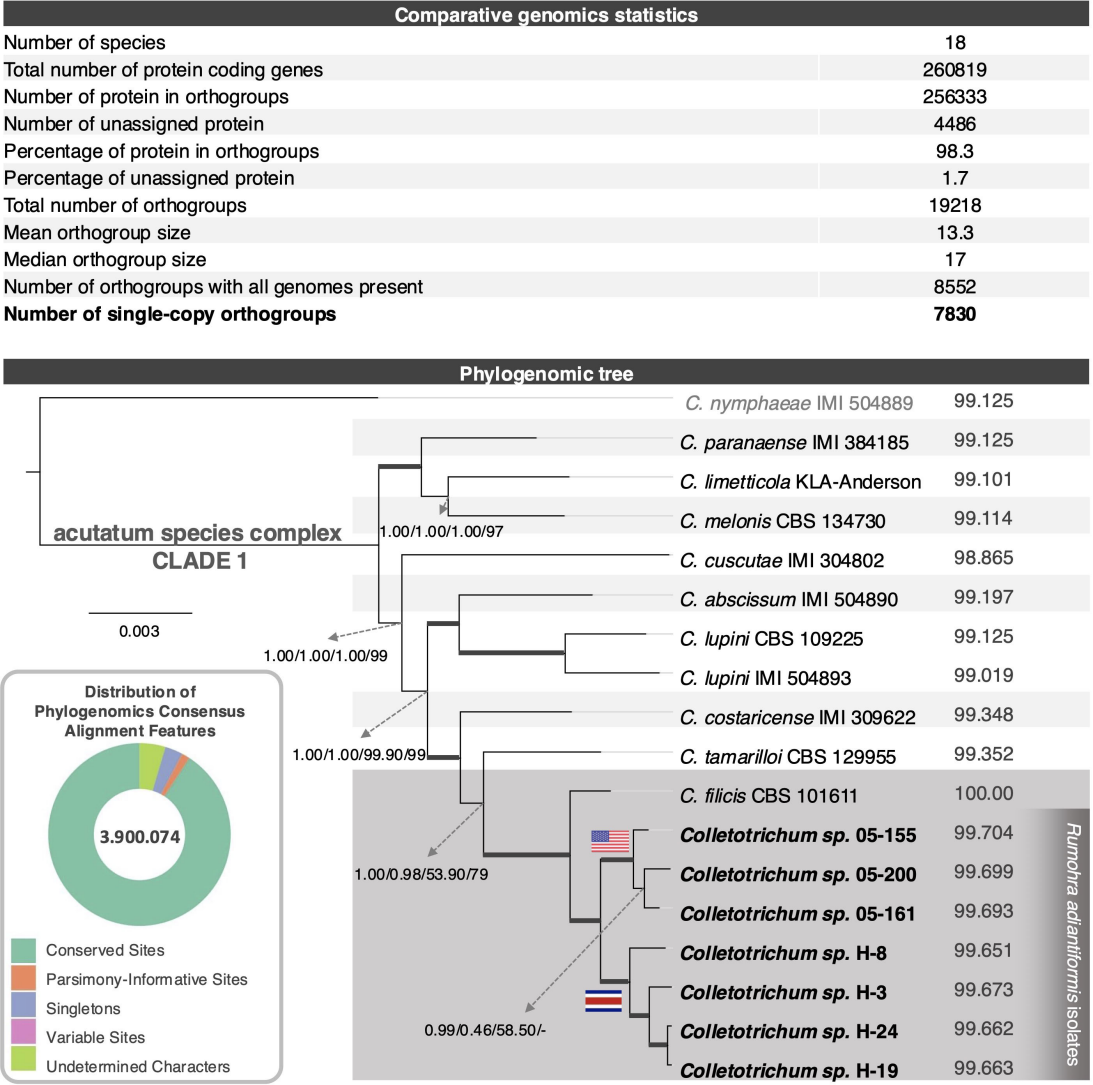
Comparative genomics and phylogenomics of selected *Colletotrichum* strains. The upper section of the figure presents key orthogroups statistics, including the total number of genes assigned to orthogroups, the total number of orthogroups identified, and the number of single-copy orthogroups shared across all genomes. The subset of single copy orthogroups used for phylogenomics is highlighted. In the lower-left section, a doughnut chart summarizes alignment features crucial for phylogenomic analyses. Conserved sites (green) dominate, reflecting high genomic conservation, while variable sites (pink), parsimony-informative sites (orange), singleton sites (blue), and undetermined characters (light green) illustrate genomic diversity and complexity. The central panel features a phylogenomic tree constructed using single-copy orthologues, showing evolutionary relationships among 18 *Colletotrichum* species. The clade containing *Colletotrichum filicis* CBS101611 and isolates associated with *Rumohra adiantiformis* is highlighted in gray. Support values from multiple phylogenetic methods (Bayesian posterior probabilities and bootstrap) are reported next to the nodes in this order, MrBayes, FastTree, RAxML and MEGA11. Strains isolated from the U.S. and those from Costa Rica are marked with their respective national flags. Isolates sequenced in this work are highlighted in bold.

The phylogenomic tree (**Figure 4**) presents a detailed depiction of the evolutionary relationships among the nine species classified within Clade 1 of the acutatum species complex, as previously described by Damm et al. (2012) and by Crous et al. (2021), based on their shared orthologous gene content. Consistent with the MLST analyses, all isolates sequenced in this study and associated with *Rumohra adiantiformis* cluster closely with *C. filicis* CBS101611. The tree’s branching structure is robustly supported by high-confidence values generated through multiple analytical approaches, including Bayesian posterior probabilities, FastTree, RAxML, and MEGA bootstrap scores, ensuring reliability in the inferred phylogenetic relationships.

Orthogroup statistics (**Figure 4**) further illuminate patterns of gene conservation and genome-specific adaptations. Across the 18 species analyzed, 260,819 genes were identified, with 256,333 genes (98.3%) assigned to orthogroups, underscoring a high degree of conservation across these genomes. Only 1.7% of the genes remained unassigned, reflecting a relatively small proportion of unique genes. A total of 19,218 orthogroups were identified, capturing the diversity and evolutionary significance of the gene content in these species. Within these orthogroups, 8,552 were found to contain genes present in all genomes, likely representing a core set of conserved genes essential for fundamental fungal functions. A smaller subset of 79 orthogroups, comprising 494 genes (0.2%), specific to individual genomes. These genome-specific orthogroups may indicate adaptations unique to particular species, potentially linked to ecological niches or pathogenicity-related traits. The high proportion of genes assigned to orthogroups (98.3%) highlights the evolutionary conservation among these fungal genomes. This conservation is also reflected in the strong support values observed in the phylogenomic tree. Although limited in number, the presence of genome-specific orthogroups offers a promising avenue for future research, particularly for uncovering unique functional traits or pathogenicity-related genes that may differentiate certain species or strains within this lineage.

**Figure 5:**
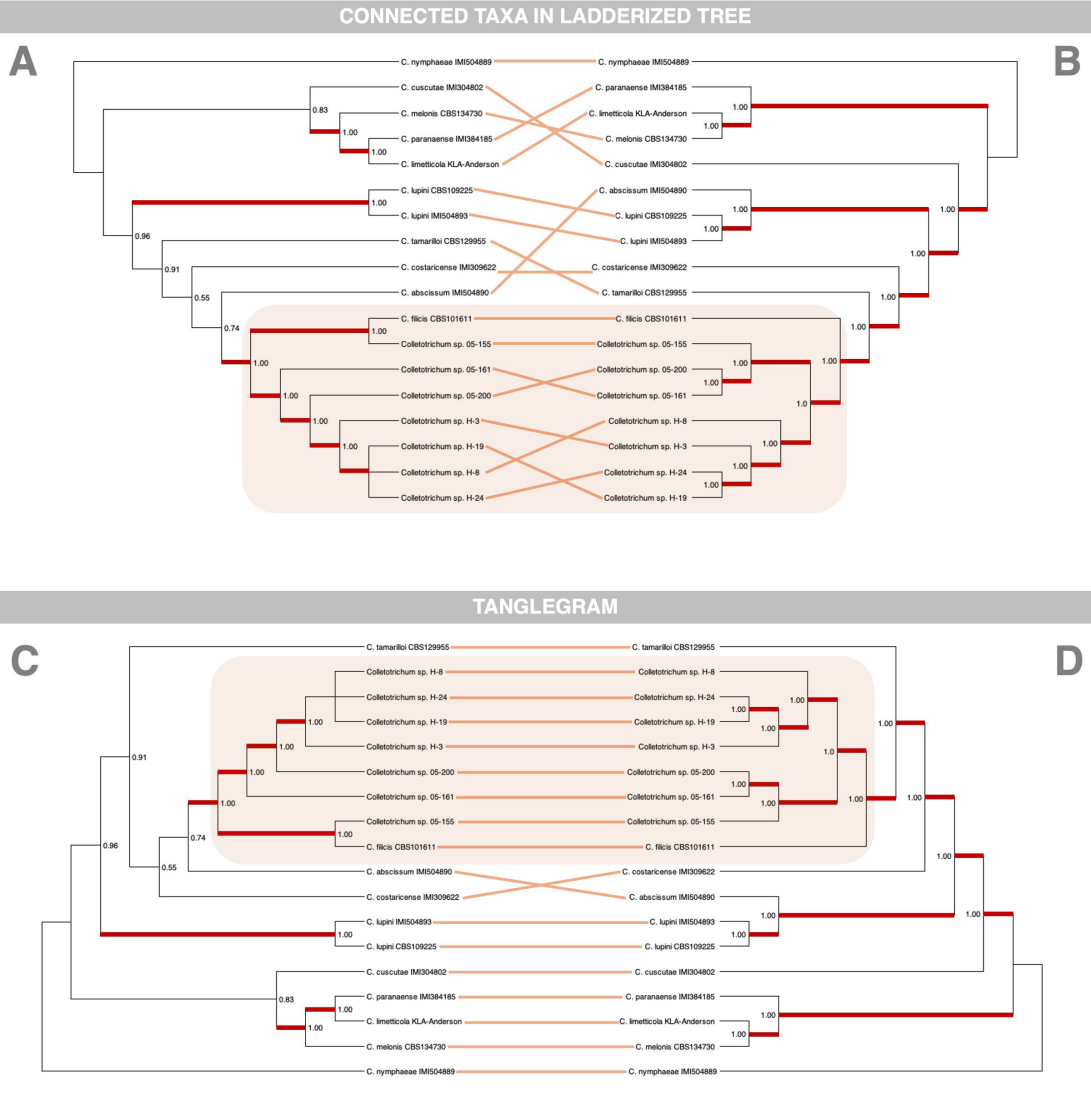
Phylogenetic comparison of *Colletotrichum* isolates using whole-genome sequencing (WGS) and multilocus sequence typing (MLST). Panels **(A, B)** present phylogenetic trees reconstructed from WGS-based and MLST-based analyses, respectively. Lines connecting corresponding taxa illustrate differences in their placement between the two methodologies, highlighting topological inconsistencies. Panels **(C, D)** display tanglegrams generated using the NN-tanglegram method in SplitsTree v6.4.12 (Huson and Bryant, 2024), which heuristically rearranges taxa to minimize line crossings while preserving the overall tree topology. Although this approach does not explicitly quantify topological incongruences, it provides a visual representation of how taxa correspond between the two phylogenies, emphasizing structural differences in their inferred relationships.

Interestingly, the phylogenomic analysis reveals notable differences in topology compared to the MLST approach, particularly in the clustering of *Rumohra adiantiformis*-associated isolates and their geographic structuring (**Figure 5**). The phylogenomic tree robustly places all *R. adiantiformis*-associated isolates together, forming a sister cluster to *C. filicis* CBS101611. This clustering is supported consistently across various methodologies, including MrBayes, FastTree, RAxML, and MEGA. By analyzing all single-copy genes conserved across genomes, the phylogenomic approach captures deeper evolutionary signals, resolving ambiguities that MLST cannot. By leveraging this extensive genomic dataset, the phylogenomic approach incorporates regions of the genome under evolutionary pressure, resulting in more precise and insightful interpretations. Additionally, the phylogenomic tree provides clearer resolution of geographic patterns. All seven isolates sequenced in this study cluster together but separately from *C. filicis* CBS101611.

Interestingly, the isolates associated with *Rumohra adiantiformis* formed two distinct clades, exhibiting a notable geographic pattern. One lineage consisted of isolates from the United States (05-161, 05-200, 05-155), while the other comprised isolates from Costa Rica (H-24, H-19, H-8, H-31) (MacKenzie et al., 2009). This geographic structuring suggests potential genetic differentiation between populations from these regions. However, this pattern was not evident in the MLST-based phylogenetic tree, where these isolates remained intermixed without clear separation (**Figure 5**).

The ability of the phylogenomic approach to detect these patterns underscores its utility in understanding population structure and evolutionary dynamics. The clustering of isolates into distinct geographic lineages offers insights into potential adaptations to local environments or host preferences. The differences observed between MLST and phylogenomic topologies emphasize the limitations of MLST in resolving complex evolutionary relationships. While MLST relies on a limited set of genes and often overlooks subtle genomic signals, the phylogenomic approach analyzes thousands of single-copy orthologues, providing enhanced clarity and reliability in discerning species relationships. These findings demonstrate the superiority of the phylogenomic method in uncovering both evolutionary history and geographic differentiation, offering a robust framework for studying *Colletotrichum* species.

## Discussion

Multilocus sequence typing (MLST) is a widely used approach for fungal species identification, forming the foundation of the genealogical concordance phylogenetic species recognition (GCPSR). This empirical method relies on the sequencing of multiple genetic *loci* combined with phylogenetic analysis to assess congruence among gene trees (Nguyen et al., 2015). GCPSR is particularly valuable for delineating species that are morphologically similar or reproduce asexually (Damm et al., 2010; Jayawardena et al., 2021). By enabling the identification of closely related taxa, GCPSR has been instrumental in resolving taxonomic ambiguities within these groups. However, its utility has limitations. For example, *C. kahawae* has been recognized as a subspecies rather than a distinct species due to challenges in distinguishing it from its closely related species, *Colletotrichum cigarro*, when using MLST with standard *loci*. This exemplifies the constraints of this method, particularly in cases involving recent evolutionary divergence, where the genetic differences may be too subtle for standard *loci* to detect effectively (Weir et al., 2012).

A real challenge in GCPSR and MLST lies in possible inconsistencies between phylogenetic trees generated from different *loci*. Single-locus and multilocus phylogenetic analyses often yield conflicting topologies, reflecting the complexity of accurately delimiting species boundaries (Gatesy and Baker, 2005; Silva et al., 2012a, b). These discrepancies could arise from the unique evolutionary trajectories and variability of individual genes but also from limitations in the alignment tools normally used. Single-locus phylogenies are inherently limited because they represent only the evolution of specific genetic regions, rather than capturing the organism’s entire genomic history (Frantz et al., 2013). As a result, MLST may struggle to provide a comprehensive view of evolutionary relationships, particularly in cases of closely related or recently diverged taxa.

For the genus *Colletotrichum*, MLST analysis commonly focuses on six standardized *loci*—ITS, GAPDH, CHS-1, ACT, HIS-3, and TUB2—for species identification (Damm et al., 2012; Jayawardena et al., 2021). While effective to a degree, this approach has proven insufficient for fully resolving species within the *gloeosporioides* complex due to the genus’s extensive diversity and complexity. Recent studies have suggested incorporating additional *loci*, such as GS and Apn2/Mat, to improve resolution in these challenging cases (Silva et al., 2012a; Liu et al., 2015; Sharma et al., 2013). Despite these enhancements, MLST remains limited in its ability to capture genome-wide evolutionary signals, making it less suitable for resolving taxonomic ambiguities in closely related or rapidly evolving fungal species.

In this study, we assess the potential of whole-genome sequencing (WGS) to resolve taxonomic ambiguities within *Colletotrichum*. We sequenced and analyzed seven strains isolated from *Rumohra adiantiformis* across various locations in North and Central America, which had previously been described but could not be reliably characterized using an MLST approach. This analysis was made possible by the availability of at least one reference genome for each described species within Clade 1 of the acutatum species complex (Damm et al., 2012; Talhinhas and Baroncelli, 2021; Baroncelli et al., 2024).

Despite the overall high quality of the genome assemblies obtained, differences in contiguity and completeness were observed among isolates. These variations may be attributed to sequencing platform limitations, differences in assembly algorithms, or intrinsic genomic complexity. Nevertheless, these data provide valuable insights into the diversity and evolutionary dynamics of the genus. Moreover, the assembled genomes serve as a robust and comprehensive resource for downstream analyses, including phylogenomics and investigations of protein-coding genes.

Overall phylogenomic analysis provided a more robust alternative to MLST by utilizing genome-wide orthologous gene content to infer evolutionary relationships. The phylogenomic tree constructed from this data offered a clear and detailed depiction of relationships among all analyzed isolates. Notably, all *Colletotrichum* isolates associated with *Rumohra adiantiformis* clustered with *C. filicis* (also originally isolated from an unidentified fern), confirming their close evolutionary relationship. Importantly, the phylogenomic tree revealed two distinct lineages within this group: one comprising isolates from Florida, USA (05-161, 05-200, 05-155) and the other from Costa Rica (H-24, H-19, H-8, H-31) (MacKenzie et al., 2009). This host and geographic separation, entirely absent in the MLST analysis, underscores the superior resolution offered by phylogenomics.

The identification of these distinct lineages holds significant implications for understanding the evolutionary history, ecological adaptations, and potential pathogenic behaviors of these isolates. Geographic or environmental factors likely drove the divergence of the Florida and Costa Rica lineages, highlighting the influence of localized evolutionary pressures. This level of resolution emphasizes the critical role of genome-wide data in uncovering evolutionary and ecological dynamics that are obscured in traditional multilocus analyses.

Despite starting with suboptimal raw data, the integration of preprocessing tools such as Trimmomatic, FLASH, and SPAdes enabled the generation of high-quality genome assemblies suitable for downstream analyses. Orthogroup detection with OrthoFinder and alignment refinement with Gblocks further ensured that the most phylogenetically informative regions were retained for tree construction. The resulting phylogenomic tree was validated by strong support values derived from multiple methods, including Bayesian posterior probabilities, FastTree, RAxML, and MEGA bootstrap analyses, providing confidence in the inferred relationships.

Overall, the phylogenomic approach outperformed MLST by offering a comprehensive genome-wide perspective, resolving evolutionary relationships with higher clarity, and revealing novel insights into the population structure of *Colletotrichum*. The clustering of isolates into two distinct geographic lineages not only demonstrates the limitations of MLST in capturing such details but also underscores the power of phylogenomics to advance fungal systematics. This study highlights the importance of adopting genome-wide approaches to address taxonomic ambiguities, particularly in complex and diverse fungal genera such as *Colletotrichum*. By enabling the identification of fine-scale evolutionary patterns and potential functional adaptations, phylogenomics offers a powerful tool for understanding the evolutionary and ecological dynamics of fungal pathogens.

## Conclusions

Our pipeline integrates whole-genome sequencing (WGS) with robust bioinformatics workflows to characterize the genetic diversity of *Colletotrichum* isolates at the genomic level. This comprehensive approach provides significantly greater resolution than traditional methods, facilitating fine-scale taxonomic analysis and uncovering evolutionary relationships with high precision.

The protocol was validated using multiple *Colletotrichum* isolates and demonstrated superior performance compared to MLST-based methods, particularly in distinguishing closely related species and subspecies. This enhanced resolution is crucial for accurate species delimitation and pathogen diagnostics, making it a valuable tool for studying cryptic species and resolving taxonomic ambiguities within the *Colletotrichum* genus.

Considering that WGS approaches are becoming increasingly cost-effective, with sequencing costs steadily decreasing, it is reasonable to anticipate that in the near future, whole-genome sequencing will become a faster, cheaper, and more practical alternative to traditional PCR-based methods. As the economic and technological barriers to WGS continue to diminish, whole-genome sequencing is likely to replace targeted PCR approaches for species identification and molecular diagnostics.

This protocol represents a significant advancement in the molecular characterization of *Colletotrichum* species. By leveraging high-throughput sequencing technologies, it improves taxonomic accuracy, enhances our understanding of evolutionary relationships, and has practical applications for disease diagnostics, crop protection, and fungal pathogen management. As sequencing technologies continue to evolve, this integrated approach will remain a cornerstone of fungal genomics research, driving progress in both fundamental and applied sciences.

## Data Availability

The datasets presented in this study can be found in online repositories. The names of the repository/repositories and accession number(s) can be found below: https://www.ncbi.nlm.nih.gov/genbank/, PRJNA1193664 PRJNA1193654 PRJNA1193647 PRJNA1193589 PRJNA1193658 PRJNA1193665 PRJNA1193671.

## Supplementary material

Supplementary Table 1 Summary of sequencing approaches, coverage, assembly methods and statistics (based on QUAST and BUSCO) for genomes used in this study.

Supplementary Table 2 Comparison of statistics (such as sequence length, alignment quality, percentage gaps, and overall composition) for concatenated alignments based on single-copy orthologous proteins and Multi-Locus Sequence Typing (MLST) approaches.

Supplementary Figure 1 The figure presents the phylogenetic trees constructed for: the internal transcribed spacer (ITS) region, a partial sequence of the glyceraldehyde-3-phosphate dehydrogenase (GAPDH) gene, the glutamine synthetase (GS) gene, the partial sequence of the beta-tubulin 2 (TUB2) gene, the histone-3 (HIS-3), the chitin synthetase gene (CHS-1) and actin (ACT) and the concatenated alignment, based on analyses performed using MrBayes **(A)**, FastTree **(B)**, and RAxML **(C)**. Branches corresponding to *Colletotrichum* spp. sequences obtained in this study are highlighted in red.
