# Supplementary material for: Can whole genome sequencing resolve taxonomic ambiguities in fungi? The case study of Colletotrichum associated with ferns
Source: Front Fungal Biol. 2025 Feb 28;6:1540469. doi: 10.3389/ffunb.2025.1540469 (PMC11906685; doi:10.3389/ffunb.2025.1540469)

ACT

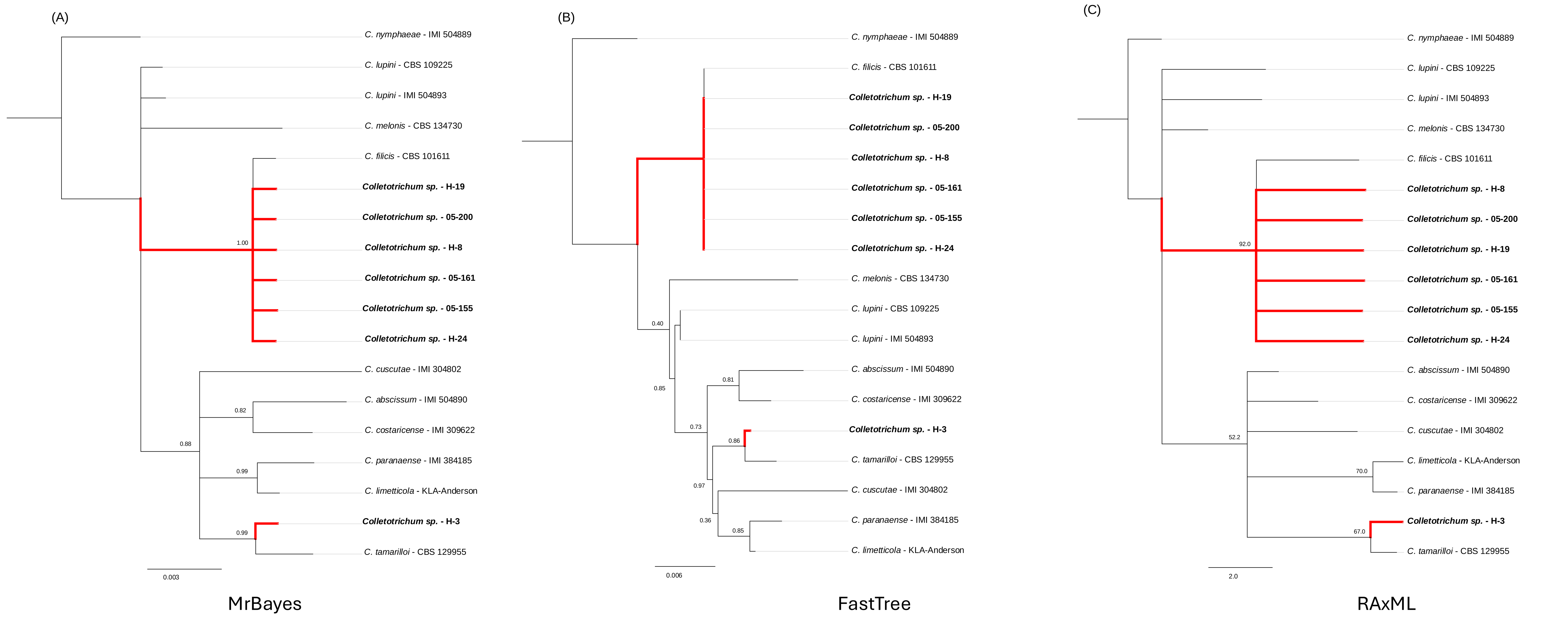

CHS-1

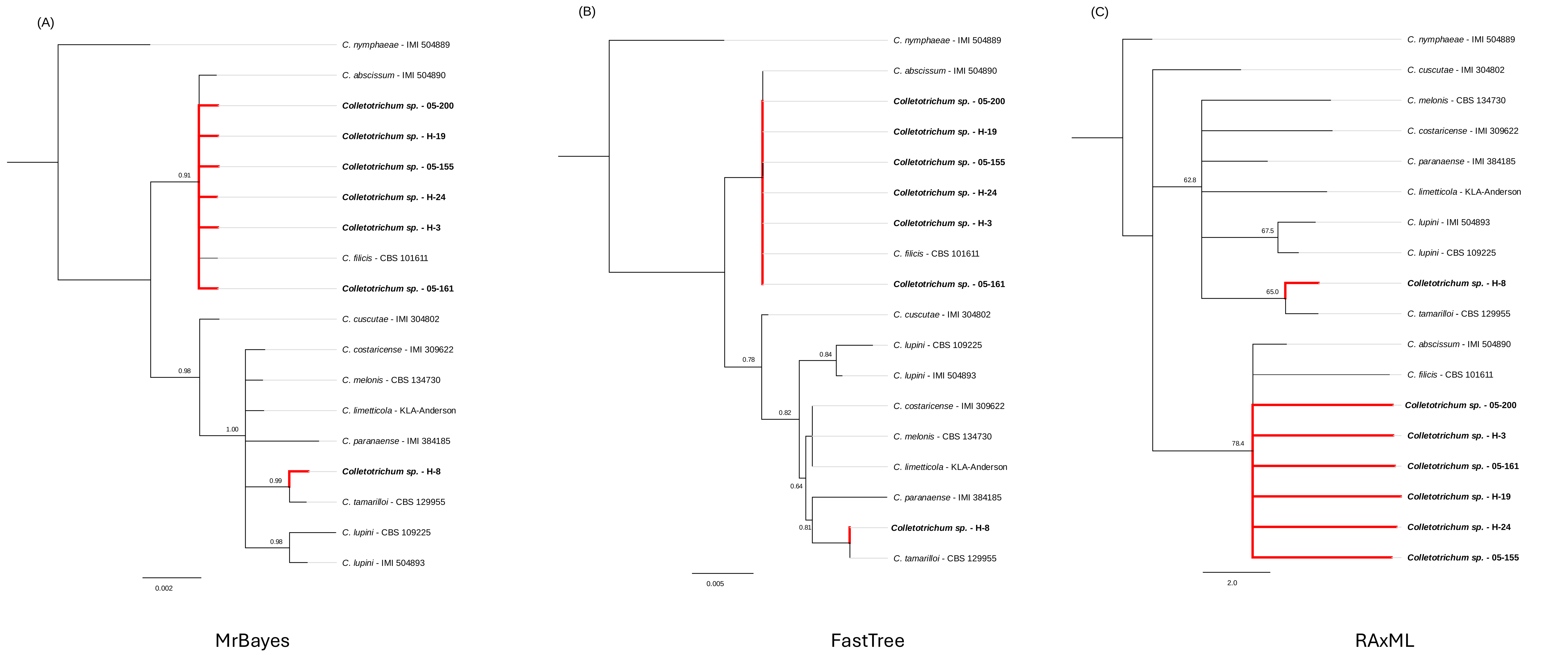

GAPDH

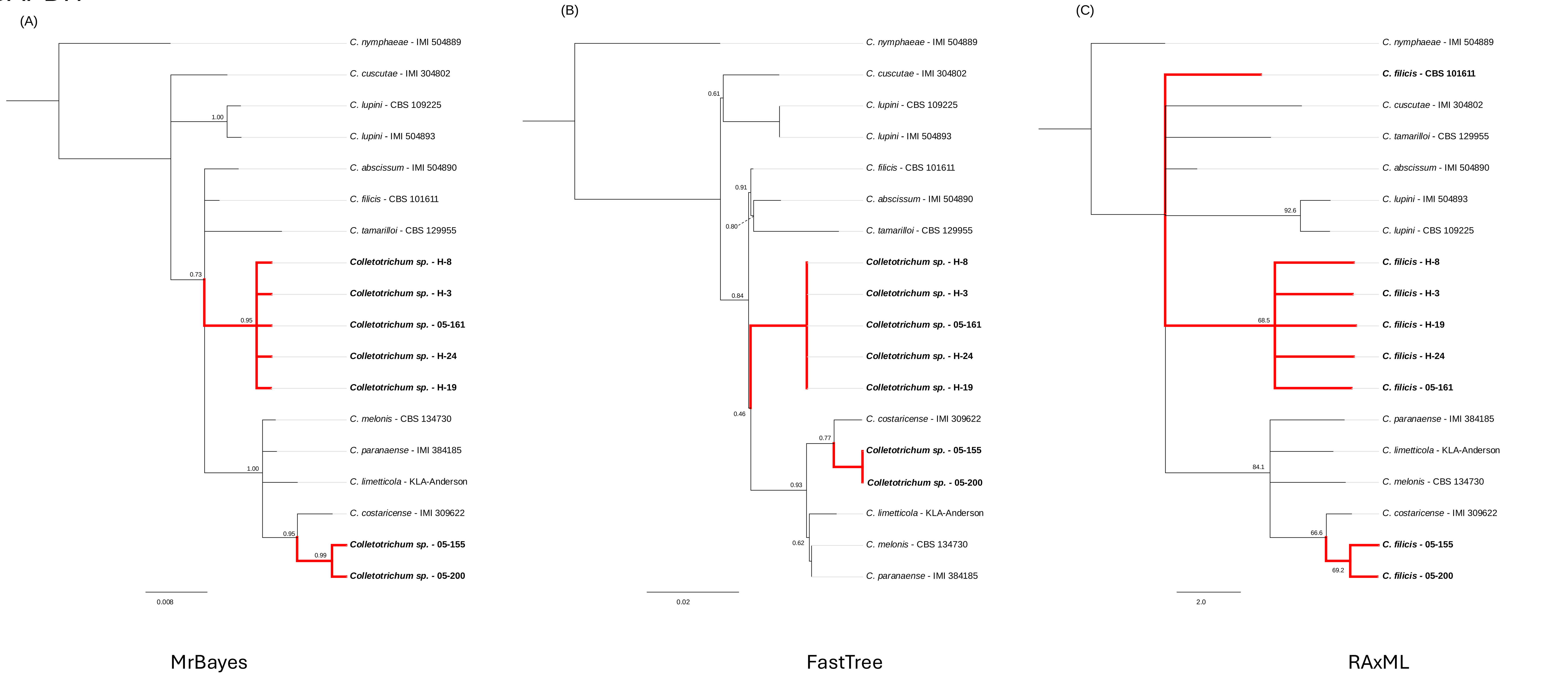

GS

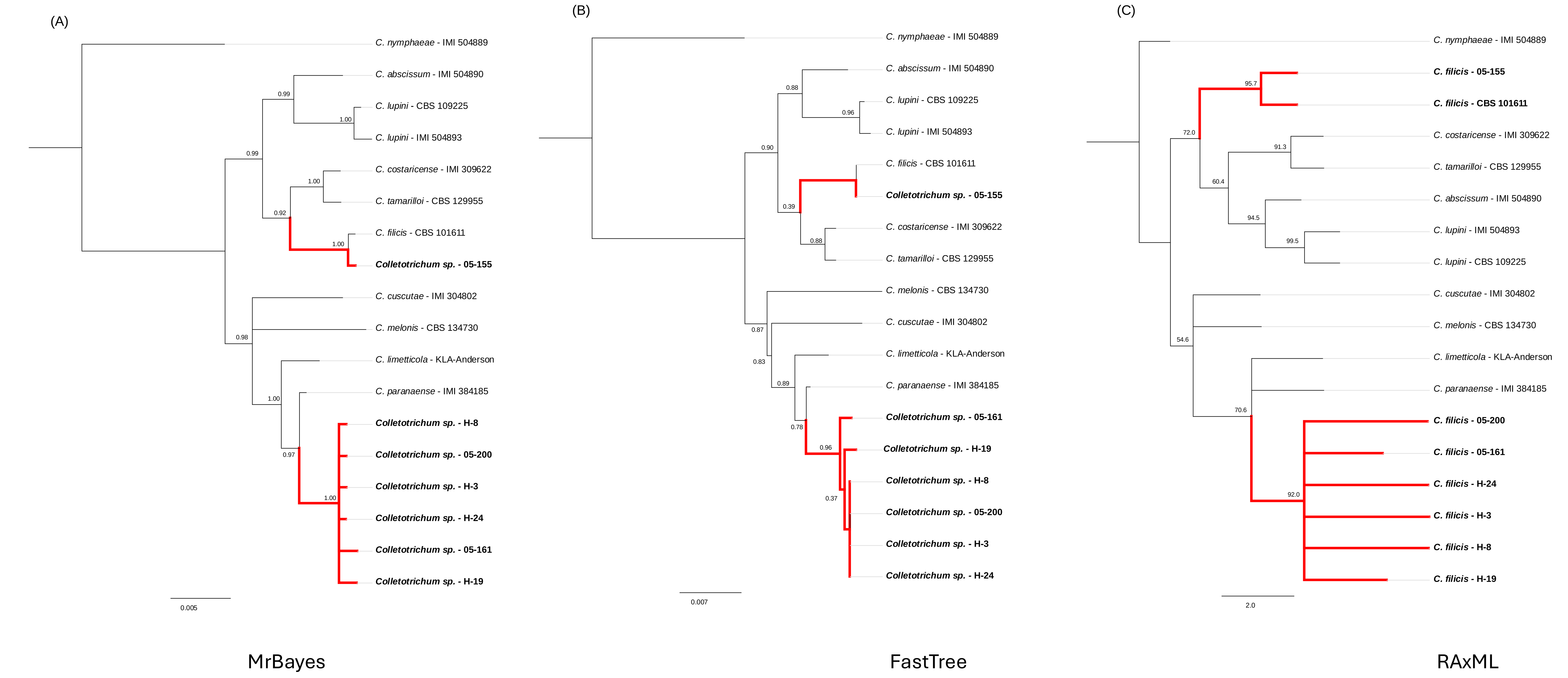
(A)
C. nymphaeae - IMI 504889
C. abscissum - IMI 504890
C. lupini - CBS 109225
C. lupini - IMI 504893
C. costaricense - IMI 309622
C. tamarilloi - CBS 129955
C. filicis - CBS 101611
Colletotrichum sp. - 05-155
C. cuscutae - IMI 304802
C. melonis - CBS 134730
C. limetticola - KLA-Anderson
C. paranaense - IMI 384185
Colletotrichum sp. - H-8
Colletotrichum sp. - 05-200
Colletotrichum sp. - H-3
Colletotrichum sp. - H-24
Colletotrichum sp. - 05-161
Colletotrichum sp. - H-19
MrBayes
(B)
C. nymphaeae - IMI 504889
C. abscissum - IMI 504890
C. lupini - CBS 109225
C. lupini - IMI 504893
C. filicis - CBS 101611
Colletotrichum sp. - 05-155
C. costaricense - IMI 309622
C. tamarilloi - CBS 129955
C. melonis - CBS 134730
C. cuscutae - IMI 304802
C. limetticola - KLA-Anderson
C. paranaense - IMI 384185
Colletotrichum sp. - 05-161
Colletotrichum sp. - H-19
Colletotrichum sp. - H-8
Colletotrichum sp. - 05-200
Colletotrichum sp. - H-3
Colletotrichum sp. - H-24
FastTree
(C)
C. nymphaeae - IMI 504889
C. filicis - 05-155
C. filicis - CBS 101611
C. costaricense - IMI 309622
C. tamarilloi - CBS 129955
C. abscissum - IMI 504890
C. lupini - IMI 504893
C. lupini - CBS 109225
C. cuscutae - IMI 304802
C. melonis - CBS 134730
C. limetticola - KLA-Anderson
C. paranaense - IMI 384185
C. filicis - 05-200
C. filicis - 05-161
C. filicis - H-24
C. filicis - H-3
C. filicis - H-8
C. filicis - H-19
RAxML

HIS-3

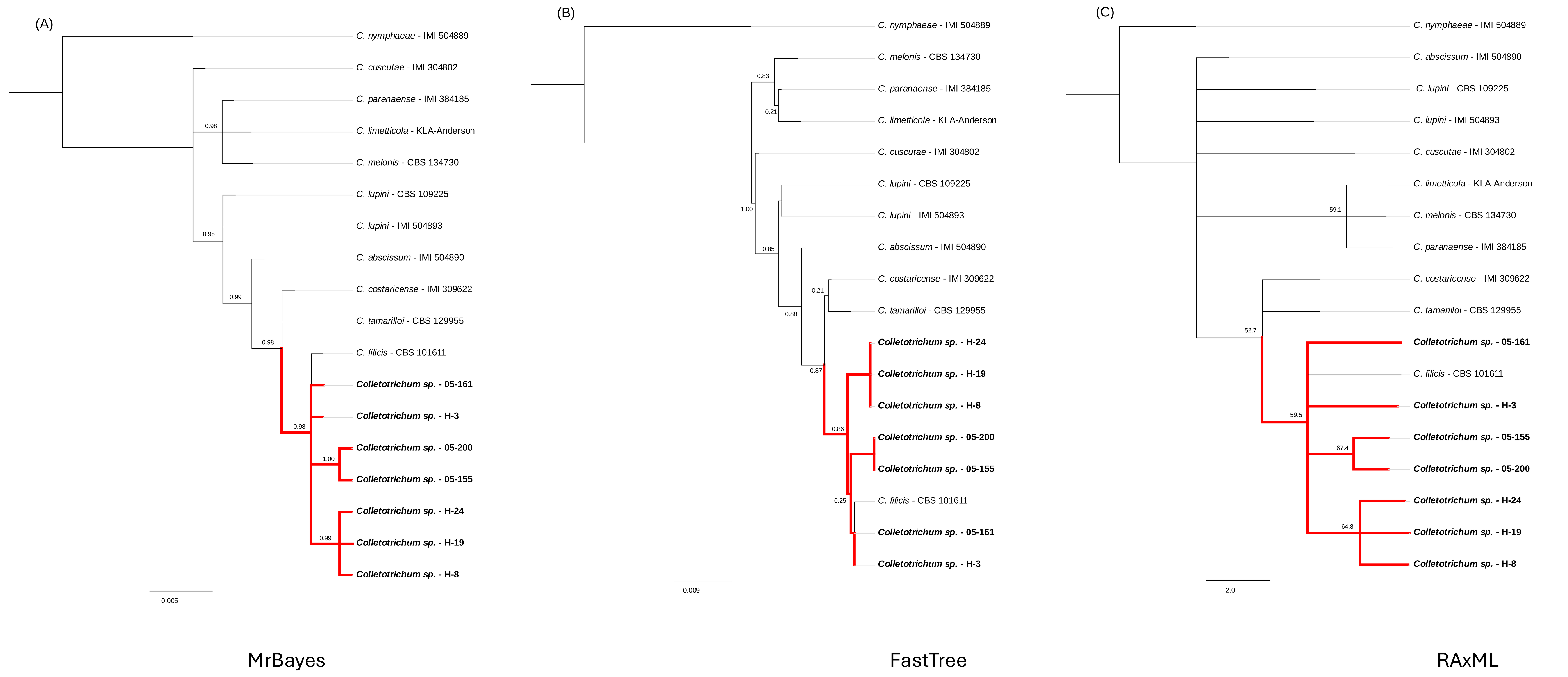
(A)
C. nymphaeae - IMI 504889
C. cuscutae - IMI 304802
C. paranaense - IMI 384185
C. limetticola - KLA-Anderson
C. melonis - CBS 134730
C. lupini - CBS 109225
C. lupini - IMI 504893
C. abscissum - IMI 504890
C. costaricense - IMI 309622
C. tamarilloi - CBS 129955
C. filicis - CBS 101611
Colletotrichum sp. - 05-161
Colletotrichum sp. - H-3
Colletotrichum sp. - 05-200
Colletotrichum sp. - 05-155
Colletotrichum sp. - H-24
Colletotrichum sp. - H-19
Colletotrichum sp. - H-8
MrBayes
(B)
C. nymphaeae - IMI 504889
C. melonis - CBS 134730
C. paranaense - IMI 384185
C. limetticola - KLA-Anderson
C. cuscutae - IMI 304802
C. lupini - CBS 109225
C. lupini - IMI 504893
C. abscissum - IMI 504890
C. costaricense - IMI 309622
C. tamarilloi - CBS 129955
Colletotrichum sp. - H-24
Colletotrichum sp. - H-19
Colletotrichum sp. - H-8
Colletotrichum sp. - 05-200
Colletotrichum sp. - 05-155
C. filicis - CBS 101611
Colletotrichum sp. - 05-161
Colletotrichum sp. - H-3
FastTree
(C)
C. nymphaeae - IMI 504889
C. abscissum - IMI 504890
C. lupini - CBS 109225
C. lupini - IMI 504893
C. cuscutae - IMI 304802
C. limetticola - KLA-Anderson
C. melonis - CBS 134730
C. paranaense - IMI 384185
C. costaricense - IMI 309622
C. tamarilloi - CBS 129955
Colletotrichum sp. - 05-161
C. filicis - CBS 101611
Colletotrichum sp. - H-3
Colletotrichum sp. - 05-155
Colletotrichum sp. - 05-200
Colletotrichum sp. - H-24
Colletotrichum sp. - H-19
Colletotrichum sp. - H-8
RAxML

ITS

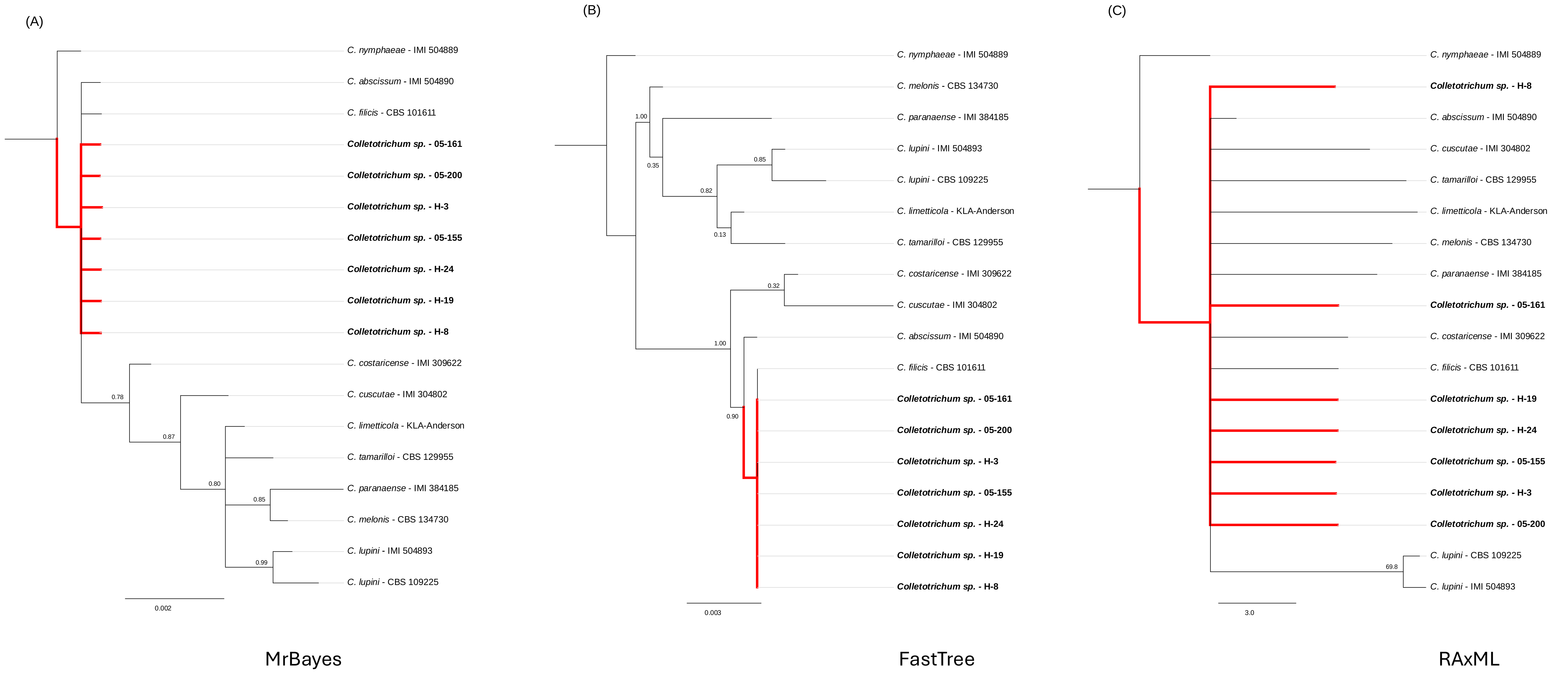
(A)
C. nymphaeae - IMI 504889
C. abscissum - IMI 504890
C. filicis - CBS 101611
Colletotrichum sp. - 05-161
Colletotrichum sp. - 05-200
Colletotrichum sp. - H-3
Colletotrichum sp. - 05-155
Colletotrichum sp. - H-24
Colletotrichum sp. - H-19
Colletotrichum sp. - H-8
C. costaricense - IMI 309622
C. cuscutae - IMI 304802
C. limetticola - KLA-Anderson
C. tamarilloi - CBS 129955
C. paranaense - IMI 384185
C. melonis - CBS 134730
C. lupini - IMI 504893
C. lupini - CBS 109225
MrBayes
(B)
C. nymphaeae - IMI 504889
C. melonis - CBS 134730
C. paranaense - IMI 384185
C. lupini - IMI 504893
C. lupini - CBS 109225
C. limetticola - KLA-Anderson
C. tamarilloi - CBS 129955
C. costaricense - IMI 309622
C. cuscutae - IMI 304802
C. abscissum - IMI 504890
C. filicis - CBS 101611
Colletotrichum sp. - 05-161
Colletotrichum sp. - 05-200
Colletotrichum sp. - H-3
Colletotrichum sp. - 05-155
Colletotrichum sp. - H-24
Colletotrichum sp. - H-19
Colletotrichum sp. - H-8
FastTree
(C)
C. nymphaeae - IMI 504889
Colletotrichum sp. - H-8
C. abscissum - IMI 504890
C. cuscutae - IMI 304802
C. tamarilloi - CBS 129955
C. limetticola - KLA-Anderson
C. melonis - CBS 134730
C. paranaense - IMI 384185
Colletotrichum sp. - 05-161
C. costaricense - IMI 309622
C. filicis - CBS 101611
Colletotrichum sp. - H-19
Colletotrichum sp. - H-24
Colletotrichum sp. - 05-155
Colletotrichum sp. - H-3
Colletotrichum sp. - 05-200
C. lupini - CBS 109225
C. lupini - IMI 504893
RAxML

TUB

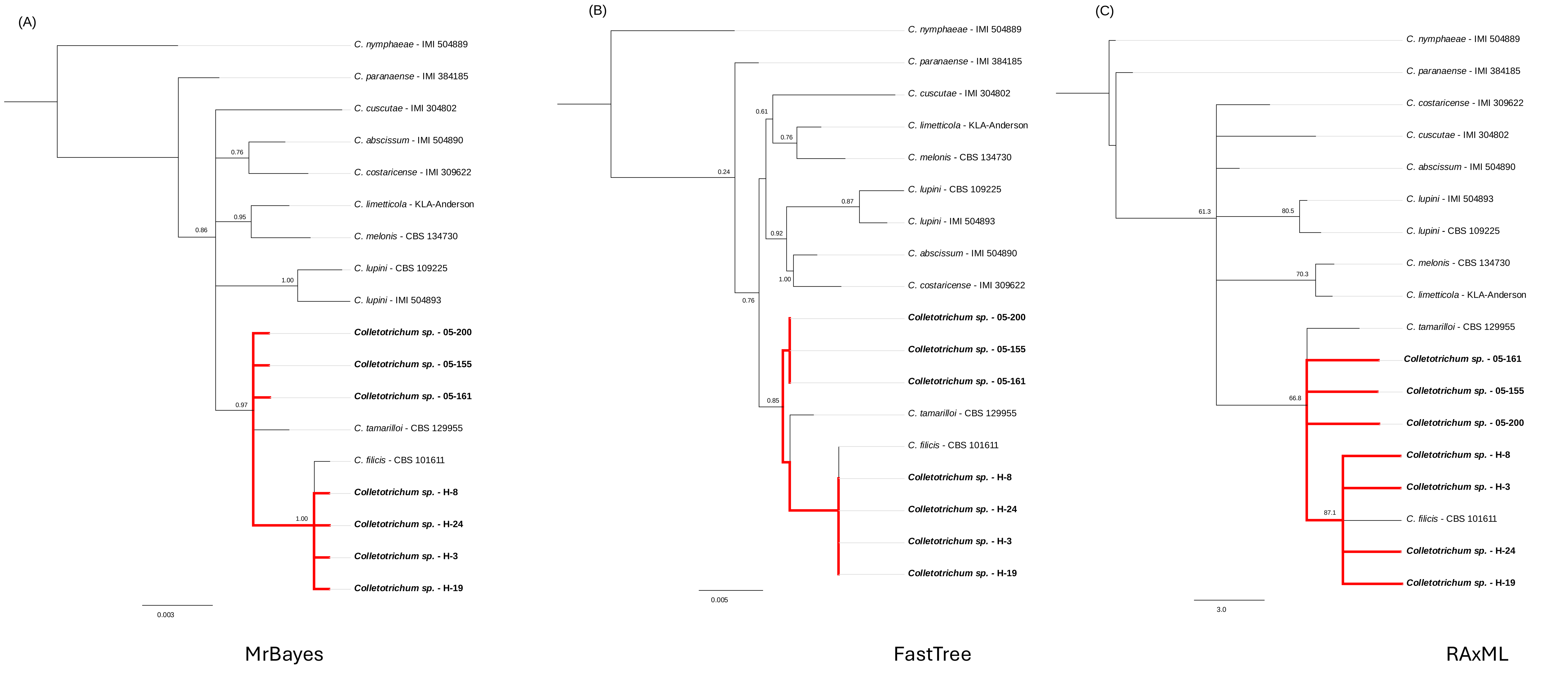

Concatenated

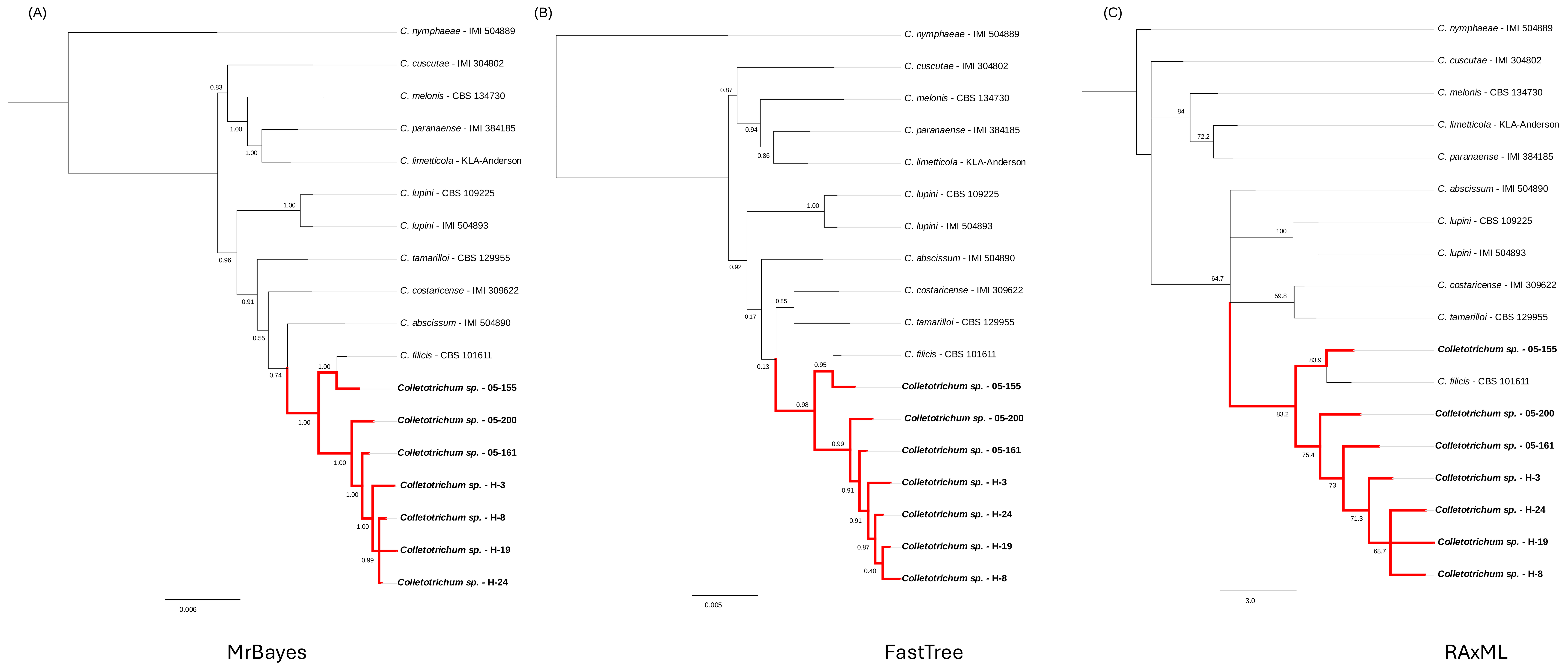

Supplement: Supplementary Figure 1 — The figure presents the phylogenetic trees constructed for: the internal transcribed spacer (ITS) region, a partial sequence of the glyceraldehyde-3-phosphate dehydrogenase (GAPDH) gene, the glutamine synthetase (GS) gene, the partial sequence of the beta-tubulin 2 (TUB2) gene, the histone-3 (HIS-3), the chitin synthetase gene (CHS-1) and actin (ACT) and the concatenated alignment, based on analyses performed using MrBayes (A), FastTree (B), and RAxML (C). Branches corresponding to Colletotrichum spp. sequences obtained in this study are highlighted in red. [file DataSheet1.pdf]
